# Risk factors in the illness-death model: Simulation study and the partial differential equation about incidence and prevalence

**DOI:** 10.1371/journal.pone.0226554

**Published:** 2019-12-17

**Authors:** Annika Hoyer, Sophie Kaufmann, Ralph Brinks

**Affiliations:** 1 Institute for Biometrics and Epidemiology, German Diabetes Center, Leibniz Center for Diabetes Research at Heinrich Heine University Düsseldorf, Düsseldorf, Germany; 2 Hiller Research Unit for Rheumatology, Heinrich Heine University Düsseldorf, Düsseldorf, Germany; International Prevention Research Institute, FRANCE

## Abstract

Recently, we developed a partial differential equation (PDE) that relates the age-specific prevalence of a chronic disease with the age-specific incidence and mortality rates in the illness-death model (IDM). With a view to planning population-wide interventions, the question arises how prevalence can be calculated if the distribution of a risk-factor in the population shifts. To study the impact of such possible interventions, it is important to deal with the resulting changes of risk-factors that affect the rates in the IDM. The aim of this work is to show how the PDE can be used to study such effects on the age-specific prevalence of a chronic disease, to demonstrate its applicability and to compare the results to a discrete event simulation (DES), a frequently used simulation technique. This is done for the first time based on the PDE which only needs data on population-wide epidemiological indices and is related to the von Foerster equation. In a simulation study, we analyse the effect of a hypothetical intervention against type 2 diabetes. We compare the age-specific prevalence obtained from a DES with the results predicted from modifying the rates in the PDE. The DES is based on 10000 subjects and estimates the effect of changes in the distributions of risk-factors. With respect to the PDE, the change of the distribution of risk factors is synthesized to an effective rate that can be used directly in the PDE. Both methods, DES and effective rate method (ERM) are capable of predicting the impact of the hypothetical intervention. The age-specific prevalences resulting from the DES and the ERM are consistent. Although DES is common in simulating effects of hypothetical interventions, the ERM is a suitable alternative. ERM fits well into the analytical theory of the IDM and the related PDE and comes with less computational effort.

## Introduction

Recently, we developed a partial differential equation (PDE) that links the age-specific prevalence of a chronic disease with the age-specific incidence and mortality rates [1]. This PDE is related to the classical illness-death model (IDM) where each subject of the population under consideration is either in the state *Healthy* (with respect to the considered disease), *Ill* or *Dead*. Using the PDE, we are able to estimate age-specific prevalences in case the incidence and mortality rates are given. Possible epidemiological applications of the PDE are, for instance, the projection of future case-numbers of a chronic disease [1] or the estimation of incidence rates from two or more cross-sectional surveys [2, 3]. Unfortunately, the PDE is currently rather unknown in context of non-communicable diseases and has only been used in a few cases. To strengthen the use of the PDE we aim to demonstrate applicability of that approach in case only population-wide epidemiological indices, as incidence or prevalence, are available. Furthermore, as it is of high interest in epidemiological research to project the effect of interventions on prevalences, we want to show how risk-factors can be incorporated in the PDE.

In order to study the effect of interventions on the future prevalence or for the projection of case-numbers, it is important to consider risk-factors (i.e., covariates) which modify the transition rates between the states in the IDM. There is a long history of estimating the impact of covariates on incidence and mortality rates, for instance, by the Cox proportional hazard model [4]. Kalbfleisch and Prentice gave an extensive introduction with example data in their popular textbook [5]. A more recent but briefer introduction is presented by Bland [6]. Typically, the effect of covariates is reported in terms of risk or rate reductions. For example, a meta-analysis about the effect of physical activity on the incidence of type 2 diabetes found that 2.5 hours per week of moderate-intensity physical activity (PA) decreased the incidence of diabetes by about 30% compared to almost no PA [7]. If the research question seeks for the effect of PA on the prevalence of diabetes, a study could compare a group without intervention to a group with intervention with respect to the prevalence. However, conducting an intervention study is sometimes lengthy, expensive and in some cases impossible due to practical problems. Computer models may be used to simulate the key aspects of a (hypothetical) intervention and estimate the resulting effects. For this, the recently developed PDE may be helpful by comparing the prevalence in the group without intervention to the intervention group where the incidence rate is modified by the intervention. A similar comparison could be made if mortality rates would be altered by an intervention. Such a group-wise comparison between an intervention group and a ‘business-as-usual’ group has performed in estimating the impact of increased active travel in the urban population of Germany [8].

Comparing two groups with and without intervention has limited value in studying the effects of population-wide interventions. If health authorities decide to promote a campaign for increased PA, typically not all persons in the population will start to increase their PA. Possibly, some persons will increase their activities and some will not. Assumed that before the campaign 30% of the population were active for 2.5 hours per week with moderate intensity, after the campaign this percentage could be increased to, say, 35%. Of course, other persons might also increase their PA, but remain below the recommended weekly 2.5 hours. This means, that the campaign is likely to change the the distribution of PA in the population. If we want to compare the prevalence of type 2 diabetes before and after the campaign, we are in a different situation than comparing two groups with the intervention entirely being effective in one group while the other group remains completely unaffected.

With a view to planning population-wide interventions, the question arises how the prevalence can be calculated if the distribution of a risk factor in the population shifts. In this work, we present two different approaches for answering this question.

First, the frequently used discrete event simulation (DES) is performed. DES generates relevant events in the illness-death model (IDM) for each individual, i.e., in our situation, the onset of disease and death with or without disease [9]. Second, prevalences are calculated based on the PDE. We illustrate and compare both methods using an example from type 2 diabetes. For this, we focus on the body mass index (BMI) as a risk factor. The relation between BMI on the incidence rate of type 2 diabetes and the mortality rates is considerably well understood from epidemiological studies [10, 11]. The aim of this work is to demonstrate applicability of both methods and compare them in estimating the impact of an intervention on the prevalence of a chronic disease. This is done for the first time based on the PDE developed by Brinks and Landwehr [1] which only needs data on population-wide epidemiological indices. Such a usage of a PDE with implications on the distribution of a modifiable risk factor is not well-known and can also be shown for the famous von Foerster equation [12] which applies to all age-structured equations of population dynamics. Unfortunately, this relationship is widely unknown to a broader audience [13], implying that the von Foerster equation is rarely used in medical and epidemiological research. Therefore, the present article gives a brief overview on the related mathematical background, corresponding equations and, finally, practical applications. It can basically be seen as guideline for assessing the impact of a potential intervention in an epidemiological context. Note that our work can be seen only as one ingredient for planning population-wide interventions. For assessing the overall impact of a population-wide intervention, many more aspects like, for example, costs, logistics, side-effects, sustainability and acceptance in the population, have to be considered.

The article is organized as follows: first, we give a brief overview on the IDM. Afterwards, the considered population and its characteristics are described. Then, the two methods for estimating the age-specific prevalences, which depend on the distribution of risk factors in the population, are introduced in detail. The relation of BMI and type 2 diabetes serves as an example application. After this, the results of the two methods are compared. Finally, in the discussion and conclusion the central findings are discussed critically.

## Materials and methods

First we introduce and review the IDM and a related partial differential equation. Then, we describe our hypothetical intervention and the target population and how the prevalences can be determined analytically and in a DES.

### Illness-death model

**Fig 1:**
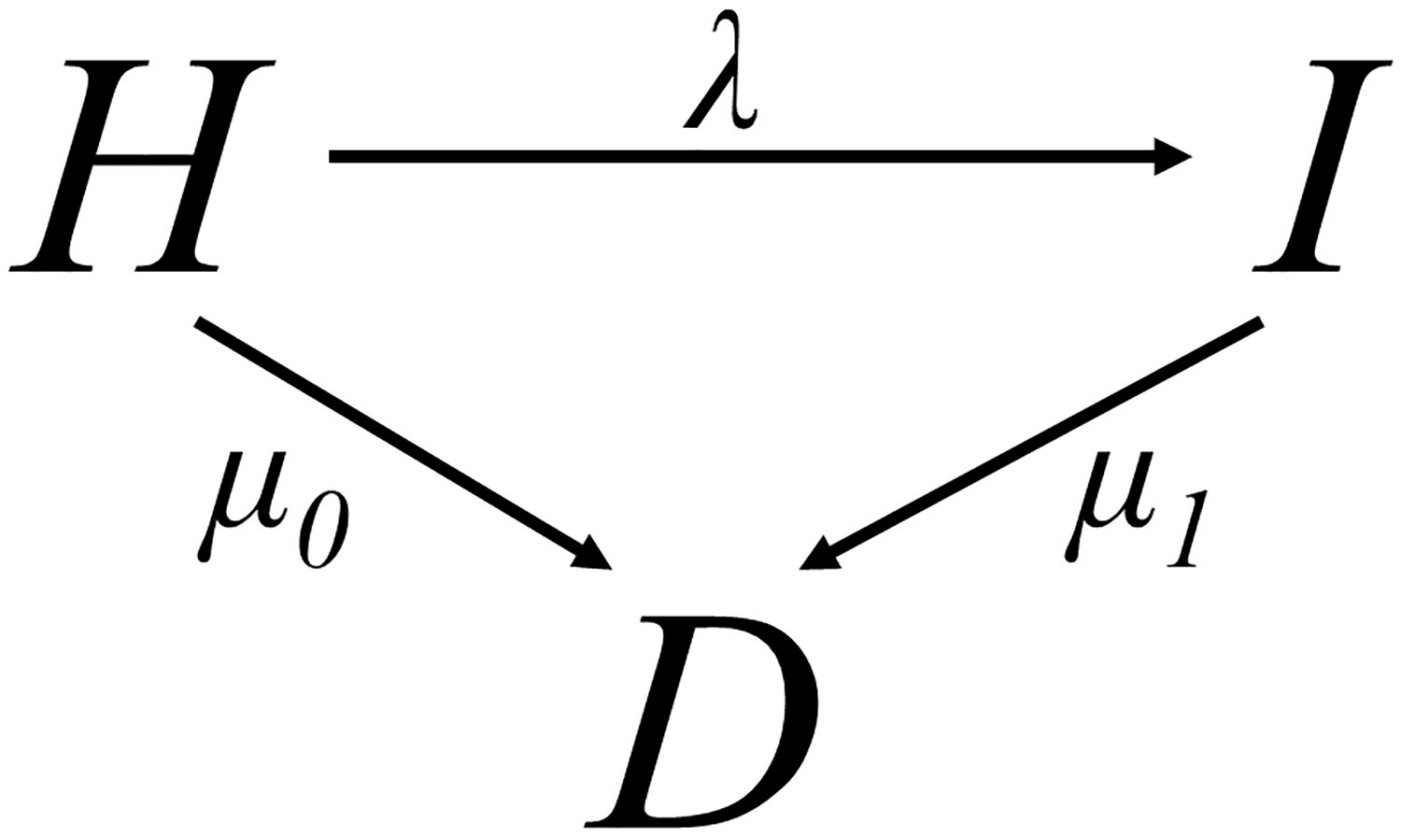
Illness-death model. The transition rates between the states *Healthy* (H), *Ill* (I), and *Dead* (D) are denoted by λ, *μ*_0_, *μ*_1_.

The classical IDM as introduced by [14] is depicted in Fig 1. The IDM consists of three states: Healthy (with respect to the considered disease (*H*)), Ill (*I*) and Dead (*D*). The transition rates between these states are the incidence rate (λ), the mortality of the susceptibles (*μ*_0_) and the mortality of the cases (*μ*_1_). These rates depend on calender time *t* and age *a*.

If we denote the prevalence of the chronic disease in those persons aged *a* at time *t* with *π*(*t*, *a*), the PDE [1] reads as

$$\begin{array}{c} (\frac{\partial }{\partial t}+\frac{\partial }{\partial a})\pi =\left(1-\pi \right)\;(\lambda -\pi \;(\mu_{1}-\mu_{0})). \end{array}$$ (1)

Given the rates λ, *μ*_0_ and *μ*_1_, the age-specific prevalence *π* = *π*(λ, *μ*_0_, *μ*_1_) can be calculated from the PDE.

### Description of the population

We simulated a birth cohort consisting of *n* = 10000 people born in 1980. Every person was followed-up from birth to death passing states presented in the IDM in Fig 1. For simplicity, we can omit the dependency on time *t* in case of a birth cohort and confine ourselves to only one time scale, age *a*, to account for temporal progression.

Although the IDM is applicable to any chronic condition, for demonstration of the methods we choose type 2 diabetes as exemplary application. As the epidemiological relationship between the BMI and type 2 diabetes is well known, we included the BMI as potential risk factor. Hence, our modelled transition rates, depend only on age *a* and the BMI [10, 11].

For each person *j*, *j* = 1, …, *n*, we assigned a BMI, denoted by *z*_*j*_, which was randomly drawn from a beta-distribution with shape parameters *ϕ* = 3 and *ψ* = 8. The support of the beta-distribution was chosen to be [17;47] which are conceivable BMI values. The choice of these parameters mirrors the right-skewed distribution of the BMI in the German male population [15]. As a high BMI is known as a potential risk factor for type 2 diabetes [11], we also simulated a population-wide intervention with the aim to reduce the BMI. This could be a lifestyle intervention as, for example, a change in eating habits or increased sporting activities. Assuming such an intervention, we generated the BMI of each person from a beta-distribution with shape parameters *ϕ* = 2.8 and *ψ* = 8. This leads to a slightly shifted distribution, meaning a change in the expected BMI from 23.9 in the group without any intervention to 23.3 in the intervention group. This seems to be a reasonable change that can be reached in a convenient amount of time as it also has been observed in the Finnish Diabetes Prevention Study [16].

**Fig 2:**
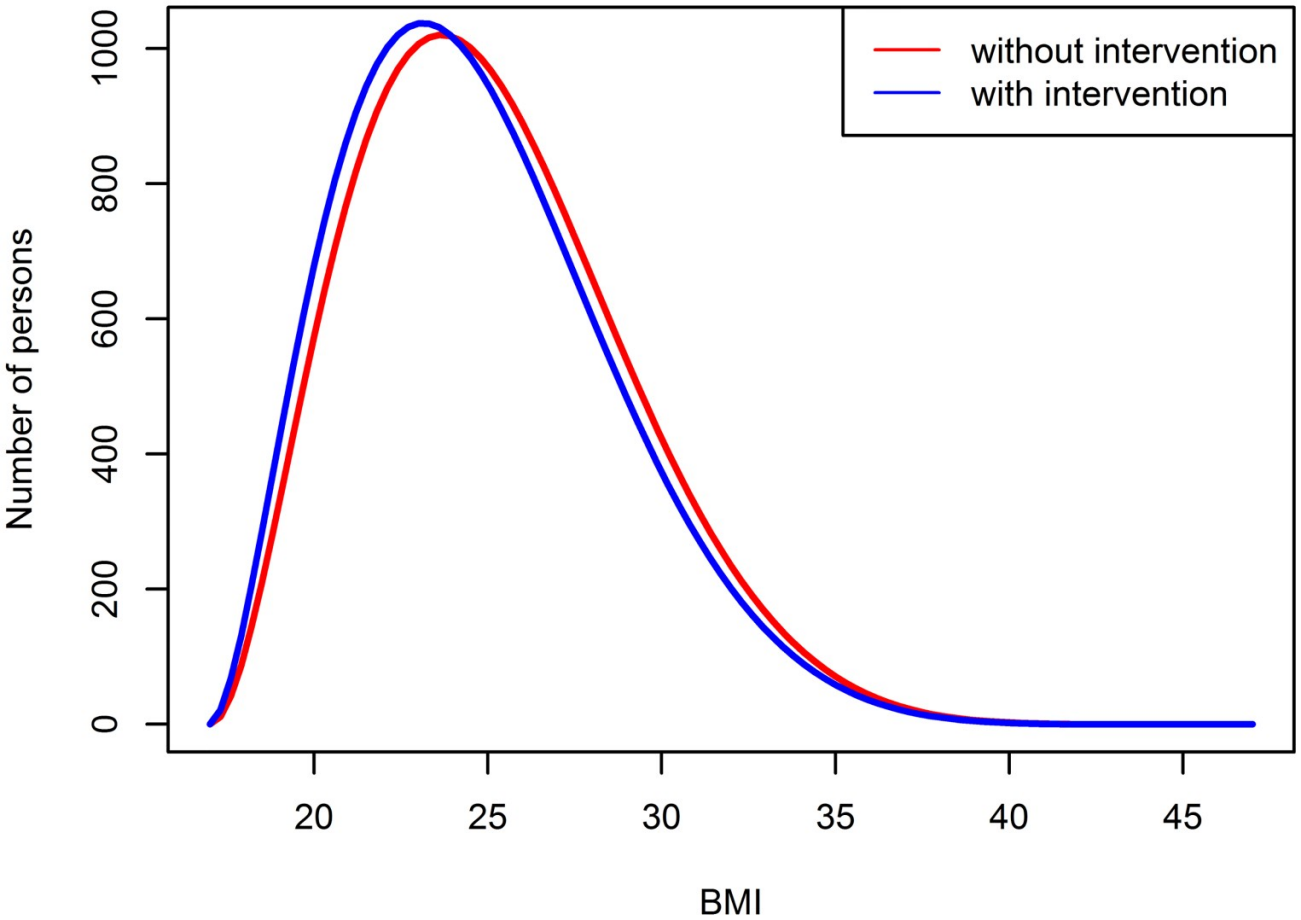
BMI distribution in Germany.

Fig 2 depicts both distributions of the BMI in the population.

### Discrete event simulation

Given the BMI *z*_*j*_ of an individual *j*, we simulated a possible transition to the diabetic state (incidence rate λ) or to the death state without contracting diabetes (mortality rate *μ*_0_). The incidence rate is chosen in accordance to the incidence rate reported by Tamayo et al. [17] for the German population. As no data on *μ*_0_ is available for Germany, we used the general mortality as given by the German Federal Statistical Office [18]. Both rates, λ and *μ*_0_, depend on the BMI as well as on age *a* as it is shown in Eqs (2) and (3):

$$\begin{array}{c} \lambda \left(a,Z\right)=\varepsilon \exp\left(\alpha a^{2}+\beta a+\gamma +\delta Z\right) \end{array}$$ (2)

and

$$\begin{array}{c} \mu_{0}\left(a\right)=\exp\left(\xi |Z-z_{0}|+\vartheta +\nu a\right), \end{array}$$ (3)

where *z*_0_ is chosen to be 23.75 according to Tobias et al. [19]. For the age-specific incidence rate, we chose an exponential parabolic function. The mortality rate *μ*_0_ was modelled using the Gompertz-Makeham law of mortality and to guarantee a j-shaped dependency on BMI, because the mortality is increasing with a high as well as with a low BMI. The parameters *ε* = 0.014, *α* = −0.001, *β* = 0.209, *γ* = −11.112 and *δ* = 0.1 for the incidence as well as *ξ* = 0.02, *ϑ* = −10.948 and *ν* = 0.095 were estimated to mimic the incidence of Tamayo et al. [17] and the general mortality in Germany, respectively.

For those individuals who contracted diabetes, we additionally simulated the mortality rate *μ*_1_ which also depends on *Z* and *a*. As it is reasonable that the mortality of people with diabetes is elevated compared to the mortality rate of people without diabetes, *μ*_1_ is assumed to be the double of *μ*_0_ as it is presented in Eq (4) [20]:

$$\begin{array}{c} \mu_{1}\left(a\right)=2\mu_{0}\left(a\right). \end{array}$$ (4)

From Eq (3), it becomes obvious that the mortality rate increases if the BMI is below or above of 23.75. As mentioned above, this is due to the empirical finding that a very low or very high BMI is associated with increased mortality rates.

For each individual *j*, we simulated failure times *T*_1*j*_ of contracting diabetes or dying without diabetes similar to Brinks et al. [21]. If the subject indexed *j* contracted diabetes, a second failure time *T*_2*j*_ was simulated, which corresponds to death after contracting the disease. It should be noted that we assume no change on the BMI in case a person contracted diabetes.

We estimated the prevalence of diabetes by dividing the number of cases at age *a* by the total number of people who are alive at age *a*. All prevalences were estimated in absence and in case of the intervention that reduces the BMI.

### Effective rate method

The DES as described in the previous section comes along with the disadvantage of being time-consuming. If many settings of input parameters have to be simulated, this can be quite cumbersome or even impossible [9]. As such, the question arises if the age-specific prevalence can be determined more time-saving and without the need for individual participant data. The effective rate method (ERM) which uses the partial differential Eq (1) offers such a possibility as this method is only based on the mean rates. In the following we present the mathematical background of the ERM as well as corresponding equations. It should be noted that these are consequences of well-known facts that also holds true for the von Foerster equation [12]. However, with respect to epidemiological applications it is helpful to reiterate. For this, we first need to convert the individual participant data from the birth cohort to mean rates.

The mean incidence rate, denoted by λ*, is calculated by the general formula

$$\begin{array}{c} \lambda^{*}=\int \lambda \left(Z\right)f\left(Z\right)dZ, \end{array}$$ (5)

where λ(*Z*) is the incidence rate for the BMI value *Z* and *f*(*Z*) is the probability density function (pdf) for the BMI. Furthermore, Eq (5) is the expected value of the incidence rate.

The same holds true for the mean mortality rates $\mu_{0}^{*}$ and $\mu_{1}^{*}$:

$$\begin{array}{c} \mu_{0}^{*}=\int \mu_{0}\left(Z\right)f\left(Z\right)dZ \end{array}$$ (6)

and

$$\begin{array}{c} \mu_{1}^{*}=HR\cdot \mu_{0}^{*}, \end{array}$$ (7)

where *HR* indicates a hazard ratio. The value of *HR* = 2 seems to be a reasonable choice [20].

As we assume the BMI to be beta-distributed, *f*(*Z*) is the pdf of this beta distribution. For the lower and upper bound of the integral, the minimum (= 17) and maximum (= 47) of the BMI in our birth cohort are chosen. Based on the mean incidence and mortality rates, we can estimate the prevalence by solving the PDE (1). In our example of a single birth cohort, we can omit the calender time *t* and the PDE simplifies to an ordinary differential Eq (8) [22]:

$$\begin{array}{c} \frac{\partial }{\partial a}\pi =\left(1-\pi \right)\left(\lambda^{*}-\pi \left(\mu_{1}^{*}-\mu_{0}^{*}\right)\right) \end{array}$$ (8)

which can be solved using the Runge-Kutta method [23] with the initial value *p*(30) = 0. This initial value is assumed to be reasonable because the majority of people contracts type 2 diabetes after the age of 30 [17].

## Results

**Fig 3:**
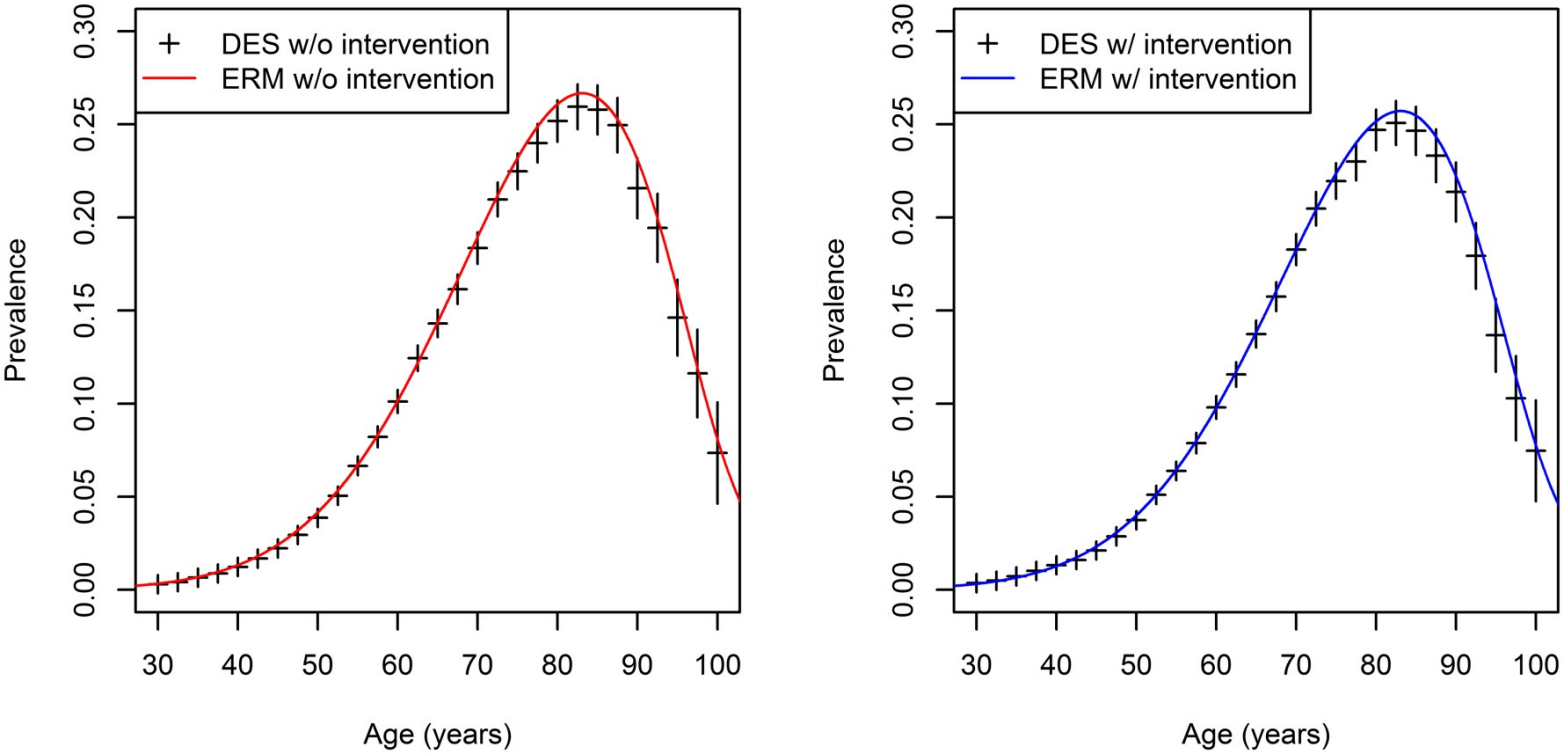
Estimated age-specific prevalences of diabetes with and without intervention using the DES and ERM. Left panel: ase-case scenario, right panel: intervention scenario.

The results of both methods are depicted in Fig 3. On the left hand side of the figure, the results for the base-case scenario (without intervention) are presented whereas the findings for the intervention scenario are presented on the right hand side. The red and blue solid lines depict the estimated prevalences based on the effective rate method. Fig 3 shows, for example, that a man aged 80 years has diabetes with a probability of approximately 25% using the DES and of 26% using the ERM in the base-case scenario. However, the prevalence of diabetes for an 80 years old man in the intervention group ranges between 24% and 25% for the DES and the ERM, respectively.

The estimated prevalences in the base-case and intervention scenario are the highest for the age group 75–90 which was also reported by Tamayo et al. [17]. However, for the population-wide intervention, the maximum prevalence is reduced from approximately 26% to 25% only by a slightly decreasing BMI. The overall prevalence of type 2 diabetes is reduced by 0.24 percentage points, from 6.51% to 6.27%. Estimates of the ERM agree very well with the point estimates of the DES method, especially in lower age groups. All of the ERM-estimates are located in the confidence intervals of the DES. The confidence intervals are somewhat wider in higher age groups above 80 years which is caused by higher mortality rates and therefore a decreased number of individuals in that age classes.

The results of the DES and the ERM agree well in the intervention case as well as in the case no intervention was assumed. Results from the DES are given as black crosses which represent the point estimates with the corresponding 95% confidence intervals. Confidence intervals for the DES result from the stochasticity of the simulation [9]. As the ERM is an exact analytical method to determine the age-specific prevalences, no confidence bounds need to be provided.

An advantageous feature of the ERM is that this method is computationally more efficient, resulting in faster simulation times. This can be seen in Table 1 where the computation times for different sample sizes, i.e. different numbers of simulated persons in the birth cohort, are given. In case of small birth cohorts of 100 persons, the DES is as fast as the ERM. However, with increasing sample sizes the DES is quite slower compared to the ERM. For example, to estimate the prevalences shown in Fig 3, the DES needs approximately 14.66 seconds whereas the ERM leads to results in just 0.36 seconds.

**Table 1:** Simulation time (in seconds) of the DES compared to the ERM.

Number of persons simulated	DES	ERM
100	0.36	0.36
1000	3.74	0.34
10000	14.66	0.36

## Discussion

In this article, we aimed to compare two methods of including the effect of a covariate into the IDM. On the one hand, a discrete event simulation was applied that simulated the relevant event times on the individual subject level. On the other hand, we integrated the effect of the covariates into the transition rates in the IDM. For this an effective rate was calculated that takes into account the distribution of the risk factor in the population (ERM). The ERM is based only on aggregated data in form of mean mortality and incidence rates. These rates are the input data for the (partial) differential equation [22] which describes the temporal and age-specific change in prevalences. For both methods, the potential effect of a covariate is modelled by a shift of the distribution of the covariate which is caused by an intervention.

We illustrated both methods using the example of type 2 diabetes mellitus and use the BMI as a very common risk factor for that non-communicable disease. Based on empirical findings, we assumed that the BMI is beta-distributed in the German population. Depending on the BMI value, individual events (contracting diabetes, dying with and without diabetes) were simulated. It becomes obvious that both methods yield similar results. This finding was confirmed in a second scenario where we changed the beta-distribution of the BMI according to a lifestyle intervention. For this work we assumed that BMI remains constant over the whole lifespan for both methods. However, in reality people will change BMI with age. Such time-varying covariates or interaction terms can be easily included in the ERM with regard to Eq (5) if data are available. However, in case of the DES it is more difficult to account for time-varying covariates or interaction terms [24]. Therefore, we decided to keep the simulation as simple as possible aiming to compare the ERM to the DES.

The ERM comes with some advantages. As it is an analytical method only mean rates are needed, i.e., the incidence and mortality rates, to calculate the prevalence at several points in time. The use of such kind of aggregated data is a definite advantage compared to the DES which can be very time consuming. Moreover, the ERM has in some cases, e.g. for non-differential mortality *m*_0_ = *m*_1_ or for given *m*_0_ and *m*_1_ [25] a solution which can be directly determined. Additionally, the ERM does automatically account for competing risks as the used differential equation is based on the IDM. In case of the DES, modelling competing events is quite more difficult and should be based on the algorithm presented in [21].

## Conclusion

Altogether, we have shown that the ERM could be used to model potential changes in covariates. Moreover, it is a time-saving alternative to the DES if we are interested in population-wide epidemiological indices like age-specific prevalences. Therefore, the ERM based on a differential equation is of high interest for future work whenever it is necessary to model the effect of covariates on the change of prevalences.

## Supporting information

S1 File Scripts for the statistical software R. The zip-file contains the complete R-code for analyzing the simulated data set and to reproduce the results. For detailed instructions unzip the file and refer to the readme.txt file.
(ZIP)

## References

[1] Brinks R, Landwehr S. Age-and time-dependent model of the prevalence of non-communicable diseases and application to dementia in Germany. Theoretical population biology. 2014;92:62–68. 10.1016/j.tpb.2013.11.006 24333220

[2] Brinks R, Landwehr S. A new relation between prevalence and incidence of a chronic disease. Mathematical Medicine and Biology. 2015;32:425–435. 10.1093/imammb/dqu024 25576933 PMC4684690

[3] Brinks R, Hoyer A, Landwehr S. Surveillance of the Incidence of Non-Communicable Diseases (NCDs) with Sparse Resources: A Simulation Study Using Data from a National Diabetes Registry, Denmark, 1995-2004. PloS one. 2016;11:e0152046 10.1371/journal.pone.0152046 27023438 PMC4811427

[4] Cox DR. Regression models and life tables (with discussion). Journal of the Royal Statistical Society. 1972;34:187–220.

[5] Kalbfleisch JD, Prentice RL. The Statistical Analysis of Failure Time Data. John Wiley & Sons; 2011.

[6] Bland M. An introduction to medical statistics. Oxford University Press (UK); 2015.

[7] Jeon CY, Lokken RP, Hu FB, van Dam RM. Physical activity of moderate intensity and risk of type 2 diabetes: a systematic review. Diabetes Care. 2007;30:744–752. 10.2337/dc06-1842 17327354

[8] Brinks R, Hoyer A, Kuss O, Rathmann W. Projected Effect of Increased Active Travel in German Urban Regions on the Risk of Type 2 Diabetes. PLoS one. 2015;10:e0122145 10.1371/journal.pone.0122145 25849819 PMC4388533

[9] Law AM. Simulation Modeling & Analysis. Fourth Edition McGraw-Hill, New York 2007.

[10] Narayan KMV, Boyle JP, Thompson TJ, Gregg EW, Williamson DF. Effect of BMI on Lifetime Risk for Diabetes in the U.S. Diabetes Care. 2007;30:1562–1566. 10.2337/dc06-2544 17372155

[11] Berentzen TL, Jakobsen MU, Halkjaer J, Tjonneland A, Sorensen TI, Overvad K. Changes in waist circumference and the incidence of diabetes in middle-aged men and women. PloS one. 2011;6:e23104 10.1371/journal.pone.0023104 21829698 PMC3150401

[12] Von Foerster H. Some remarks on changing populations In: The Kinetics of Cellular Proliferation, Stohlman F. Jr., editor. Greene and Stratton, New York.

[13] Chubb MC, Jacobsen KH. Mathematical modeling and the epidemiological research process. European Journal of Epidemiology. 2010;25:13–19. 10.1007/s10654-009-9397-9 19859816

[14] Keiding N. Age-specific incidence and prevalence: a statistical perspective. Journal of the Royal Statistical Society. Series A (Statistics in Society). 1991;371–412. 10.2307/2983150

[15] Robert Koch-Institut and Destatis. Distribution of the population to groups in terms of body mass index in percent. Classification: years, Germany, age, sex, body mass index. 2003. Available at: http://www.gbe-bund.de/gbe10/trecherche.prc_them_rech?tk=5800&tk2=6000&p_uid=gast&p_aid=10171277&p_sprache=D&cnt_ut=11&ut=6150 (Last accessed May 15, 2019).

[16] Lindström J, Louheranta A, Mannelin M, Rastas M, Salminen V, Eriksson J, Uusitupa M, Tuomilehto J, Finnish Diabetes Prevention Study Group. The Finnish Diabetes Prevention Study (DPS): Lifestyle intervention and 3-year results on diet and physical activity. Diabetes Care. 2003;26:3230–3236. 14633807 10.2337/diacare.26.12.3230

[17] Tamayo T, Brinks R, Hoyer A, Kuss O, Rathmann W. The Prevalence and Incidence of Diabetes in Germany. Deutsches Arzteblatt International. 2016;133:177–182. 10.3238/arztebl.2016.0177 PMC4850517 27118665

[18] Statistisches Bundesamt. Sterbetafel 2012/2014—Methoden- und Ergebnisbericht zur laufenden Berechnung von Periodensterbetafeln für Deutschland und die Bundesländer. Available at: https://www.destatis.de/DE/Themen/Gesellschaft-Umwelt/Bevoelkerung/Sterbefaelle-Lebenserwartung/_inhalt.html#sprg233418 (Last accessed May 15, 2019).

[19] Tobias DK, Pan A, Jackson CL, O’Reilly EJ, Ding EL, Willett WC, Manson JE, Hu FB. Body-mass index and mortality among adults with incident type 2 diabetes. New England Journal of Medicine. 2014;370:233–244. 10.1056/NEJMoa1304501 24428469 PMC3966911

[20] Saydah SH, Loria CM, Eberhardt MS, Brancati FL. Subclinical states of glucose intolerance and risk of death in the U.S. Diabetes Care. 2001;24:447–453. 10.2337/diacare.24.3.447 11289466

[21] Brinks R, Landwehr S, Fischer-Betz R, Schneider M, Giani G. Lexis Diagram and Illness-Death Model: Simulating Populations in Chronic Disease Epidemiology. PLoS one. 2014;9:e106043 10.1371/journal.pone.0106043 25215502 PMC4162544

[22] Brinks R, Landwehr S, Icks A, Koch M, Giani G. Deriving age-specific incidence from prevalence with an ordinary differential equation. Statistics in Medicine. 2013;32:2070–2078. 10.1002/sim.5651 23034867

[23] Dahlquist G, Björk A. Numerical Methods. Prentice-Hall Inc. Englewood Cliffs, New Jersey 1964.

[24] Brennan A, Chick SE, Davies R. A taxonomy of model structures for economic evaluation of health technologies. Health Economics. 2006;15:1295–1310. 10.1002/hec.1148 16941543

[25] Brinks R, Landwehr S. Change rates and prevalence of a dichotomous variable: simulations and applications. PloS one. 2015;10:e0118955 10.1371/journal.pone.0118955 25749133 PMC4352043

